# Nutrition for Sarcopenia

**DOI:** 10.14740/jocmr2361w

**Published:** 2015-10-23

**Authors:** Hidekatsu Yanai

**Affiliations:** Department of Internal Medicine, National Center for Global Health and Medicine Kohnodai Hospital, 1-7-1 Kohnodai, Chiba 272-8516, Japan. Email: dyanai@hospk.ncgm.go.jp

**Keywords:** Beta-hydroxy-beta-methylbutyrate, Energy restriction, Leucine, Protein intake, Sarcopenia

## Abstract

Aging-related sarcopenia means that muscle mass, strength, and physical performance tend to decline with age, and malnutrition is associated with sarcopenia. Therefore, nutritional interventions may make an important contribution to prevent the development of sarcopenia. Here I reviewed published articles about the effects of nutritional factors on sarcopenia in elderly people. A growing body of evidence suggests that metabolic factors associated with obesity and diabetes induce the progression of sarcopenia. However, the effectiveness and safety of caloric restriction for sarcopenia remained unclear. Protein intake and physical activity are the main anabolic stimuli for muscle protein synthesis. As optimal dietary protein intake, 1.0 - 1.2 g/kg (body weight)/day with an optimal repartition over each daily meal or 25 - 30 g of high quality protein per meal were recommended to prevent sarcopenia, which was supported by some observational studies. Protein supplementation using cheese and milk protein, essential amino acids, leucine, beta-hydroxy-beta-methylbutyrate and vitamin D has been investigated as a potential supplement to improve muscle quality in sarcopenic elderly people.

## Introduction

The number of elderly people has been rapidly increasing in Japan. The growing number of elderly people has compelled us to focus on aging-related sarcopenia. Sarcopenia indicates that muscle mass, strength, and physical performance tend to decline with age, therefore sarcopenia becomes increasingly prevalent with age [1-3]. As sarcopenia is a major predictor of frailty, hip fracture, disability and mortality in elderly persons [1-3], the development of effective management to treat it is eagerly awaited. Malnutrition is one of factors which induce sarcopenia [3]. Nutritional interventions may make an important contribution to avenues for prevention of sarcopenia. However, nutritional management for sarcopenia remains largely unknown. Here I reviewed published articles about the effects of nutritional factors on sarcopenia in elderly people.

## The Association Between Energy Intake and the Development of Sarcopenia

A growing body of evidence suggests that metabolic factors associated with obesity and diabetes induce the progression of sarcopenia [4]. Recently, the age-related change in the body composition such as a combination of excess weight and reduced muscle mass/strength is defined as sarcopenic obesity [5]. Sarcopenic obesity is associated with the upregulation of inflammatory cytokines [5], which further deteriorates sarcopenia [6]. Is energy restriction (ER) effective to prevent obesity/diabetes-related sarcopenia? In the systematic review using 52 studies including exercise (EX), ER, or ER + EX groups, 81% and 39% of the ER and ER + EX groups, respectively, lost over 15% of body weight as fat-free mass (FFM) [7]. Study participants had to have a mean age of ≥ 50 years and mean body mass index (BMI) of ≥ 25 kg/m^2^. Studies that had very low calorie diets (≤ 800 calories per day) were excluded. This study suggests that EX preserves FFM after moderate ER, and also indicates that ER induces FFM loss even in obese people. Normandin et al identified 19 published papers from 10 randomized controlled trials (RCTs) ranging from 3 to 18 months that reported independent effects of a caloric restriction (CR) on the health benefits in adults with a mean age of ≥ 65 years [8]. They concluded that the risk-to-benefit ratio of CR for the treatment of obesity in elderly adults remains unclear. We should determine the adequate energy intake to prevent or treat sarcopenia by performing further studies in the future.

## The Association Between Protein Intake and the Development of Sarcopenia

In the cross-sectional study combined five datasets (n = 900), 77% of all participants had lower than recommended protein intake [9], indicating the existence of insufficient protein intake in heterogeneous elderly populations.

Protein intake and physical activity are the main anabolic stimuli for muscle protein synthesis. Aging causes loss of various anabolic signals to muscle that are present in young people [10]. Such “anabolic resistance” is associated with the development of sarcopenia [10]. Amino acids alone stimulate muscle protein synthesis in the elderly. However, mixed nutritional supplementation failed to improve muscle mass. Volpi et al found that the response of muscle protein anabolism to hyperaminoacidemia with endogenous hyperinsulinemia is impaired in healthy elderly people [11].

How much protein should elderly people with anabolic resistance ingest? Rizzoli reported the recommendations for optimal dietary protein intake are daily 1.0 - 1.2 g/kg (body weight) with an optimal repartition over each daily meal to prevent sarcopenia [12]. Paddon-Jones et al proposed a dietary plan that includes 25 - 30 g of high quality protein per meal as dietary protein recommendations for the prevention of sarcopenia [13].

Bopp et al investigated the association between dietary protein intake and loss of lean mass (LM) during a 20-week weight loss intervention in postmenopausal women [14]. Protein intake averaged 0.62 g/kg/day (0.47 - 0.8 g/kg/day). Participants who consumed higher amounts of dietary protein lost less LM; however, these participants (0.8 g/kg/day) also lost LM. These results suggested that an inadequate protein intake may be associated with LM loss, and daily protein intake ≤ 0.8 g/kg was not sufficient to prevent LM loss in postmenopausal women.

Houston et al studied to determine the association between dietary protein and changes in total LM in elderly, community-dwelling men and women [15]. Energy-adjusted protein intake was associated with 3-year changes in LM. Participants in the highest quintile of protein intake (1.2 g/kg/day) lost approximately 40% less LM than did those in the lowest quintile of protein intake (0.8 g/kg/day).

In Japan, Kobayashi et al examined the association of protein and amino acid intake with frailty among elderly Japanese women [16]. Subjects categorized to the third (69.8 - 76.1 g/day), fourth (76.1 - 84.3 g/day), and fifth quintiles (76.1 - 84.3 g/day) of total protein intake showed significantly lower odds ratios than those to the first quintile (≤ 62.9 g/day).

Recently the study was undertaken to evaluate differences in protein intake in women with or without sarcopenia [17]. Elderly women older than 65 years with sarcopenia (n = 35) and without sarcopenia (n = 165) participated in the study. Muscle mass was significantly higher in women who had protein intake > 1.2 g/kg/day. Protein and energy intake were significant predictors of muscle mass.

These results agreed with the recommendation for protein intake such as 1.0 - 1.2 g/kg/day (daily 60 - 72 g for 60 kg weighted subjects) and 25 - 30 g of high quality protein per meal (daily 75 - 90 g).

## Clinical Trials to Study Effects of Nutritional Factors on Sarcopenia

Clinical trials to study effects of nutritional factors on sarcopenia were shown in Table 1 [18-36]. The adding daily 210 g of ricotta cheese to the habitual diet improved the markers of sarcopenia in subjects without a pronounced loss of skeletal muscle mass [18]. Dietary milk protein supplementation improved physical performance, but did not increase skeletal muscle mass in frail elderly people [19]. Fast-digestive soluble milk protein supplement for 10 days overcame muscle anabolic resistance and improved postprandial muscle protein synthesis in healthy elderly men [20]. In 65 sarcopenic elderly Malays aged 60 - 74 years, there was an increase in FFM (+5.7%) in the EX group after 12 weeks. The highest increments in lower body strength was observed in the protein supplementation group (73.2%) [21].

**Table 1:** Clinical Trials to Study Effects of Protein, Amino Acids, Leucine, Vitamin D, HMB Intake on Sarcopenia

Authors	Subjects	Study design	Resuts/Conclusions
Aleman-Mateo et al [18]	Patients with sarcopenia, ≥ 60 years (n = 40)	The intervention group received 210 g/day of ricotta cheese plus the habitual diet, while the control group followed the habitual diet with no additional intervention for 3 months.	The adding daily 210 g of ricotta cheese to the habitual diet improved the markers of sarcopenia in subjects without a pronounced loss of skeletal muscle mass.
Tieland et al [19]	Frail elderly people (n = 65)	Subjects were randomly allocated to either daily protein or placebo supplementation (15 g milk protein at breakfast and lunch), for 24 weeks.	Dietary milk protein supplementation improved physical performance, but did not increase skeletal muscle mass in frail elderly people.
Walrand et al [20]	Healthy elderly men (71.8 ± 2.4 (mean ± SD) years, n = 31)	Adequate-protein or high-protein diet together with the protein source as caseins or soluble milk proteins was provided for 10 days.	Fast-digesting soluble milk proteins improved postprandial muscle protein synthesis in elderly subjects.
Shahar et al [21]	Sarcopenic elderly Malays aged 60 - 74 years (n = 65)	Subjects were assigned to the control group, exercise group, protein supplementation group, or the combination of exercise and protein supplementation group for 12 weeks.	The exercise program improved muscle strength and body composition, while protein supplementation reduced body weight and increased upper body strength.
Kim et al [22]	Sarcopenic women aged 75 and older (n = 155)	Subjects were randomly assigned to exercise and AAS (n = 38), exercise (n = 39), AAS (n = 39), or HE (n = 39). The exercise group attended a 60-min comprehensive training program twice a week, and the AAS group ingested 3 g of a leucine-rich EAA mixture twice a day for 3 months.	Walking speed significantly increased in all three intervention groups, leg muscle mass in the exercise + AAS and exercise groups, and knee extension strength only in the exercise + AAS group. The odds ratio for leg muscle mass and knee extension strength improvement was more than four times as great in the exercise + AAS group as in the HE group.
Dillon et al [23]	Elderly women (68 ± 2 years) (n = 14)	Subjects were assigned to receive either placebo (n = 7), or 15 g EAA/day (n = 7) for 3 months.	EAA improved LM and basal muscle protein synthesis in elderly individuals.
Solerte et al [24]	Elderly subjects (66 - 84 years) with sarcopenia (n = 41)	Subjects were assigned to EAA and placebo. EAA treatment consisted of 8 g of EAA snacks twice a day.	Significant increases in whole-body LM in all areas were seen after 6 months and more consistently after 18 months of oral nutritional supplementation with EAA.
Scognamiglio et al [25]	Elderly subjects (> 65 years) with reduced physical activity (n = 100)	Subjects were randomized to receive an oral amino acids mixture (12 g/day) or placebo for 3 months.	An oral amino acids supply improved ambulatory capacity, maximal isometric muscle strength in elderly subjects.
Borsheim et al [26]	Glucose intolerant subjects (67.0 ± 5.6 years, seven females, five males)	Subjects ingested 11 g of a nutritional supplement containing EAA + arginine twice a day, between meals for 16 weeks.	Supplementation of the diet with EAA + arginine improved LM, strength and physical function.
Coker et al [27]	Elderly individuals (n = 12)	Caloric restriction diet utilizing equivalent caloric meal replacements (800 kcal/day): 1) EAAMR or 2) CMR in conjunction with 400 kcal of solid food that totaled 1,200 kcal/day designed to induce 7% weight loss.	Both groups lost about 7% of total body weight. While EAAMR did not promote a significant preservation of LM, the reduction in adipose tissue was greater in EAAMR compared to CMR.
Bukhari et al [28]	Elderly women (66 ± 2.5 years; n = 8/group)	Whey protein or novel low-dose leucine-enriched EAA (3 g, 40% leucine), single-dose administration.	There was no difference in muscle anabolism between whey protein and novel low-dose leucine-enriched EAA.
Cangussu et al [29]	Postmenopausal women (50 - 65 years) with a history of falls (n = 160)	Subjects were randomized into vitamin D group consisting of patients receiving vitamin D 1,000 IU/day orally (n = 80) or placebo group (n = 80) for 9 months.	In the vitamin D group, there was significant increase in muscle strength (+25.3%) of the lower limbs by chair rising test. In the placebo group, there was considerable loss (-6.8%) in LM.
Verschueren et al [30]	Institutionalized elderly females aged over 70 years (mean age 79.6 years) (n = 113)	In a 2 × 2 factorial-design trial, subjects were randomly assigned either to a whole-body vibration or a no-training group, receiving either a conventional dose (880 IU/day) or a high dose (1,600 IU/day) of vitamin D.	After 6 months of treatment, dynamic muscle strength improved significantly in all groups. A higher dose of vitamin D did not provide additional musculoskeletal benefit compared with conventional doses.
Daly et al [31]	Women aged 60 - 90 years who were residing in 15 retirement villages (n = 100)	Subjects were allocated to receive lean red meat (about 160 g cooked) to be consumed 6 days/week or control (1 serving pasta or rice/day) for 4 months. All women undertook resistance training 2 times/week and received 1,000 IU vitamin D/day.	A protein-enriched diet equivalent to 1.3 g/kg/day achieved through lean red meat was safe and effective for enhancing the effects of progressive resistance training on LM and muscle strength.
Bauer et al [32]	Sarcopenic primarily independent-living elderly adults (n = 380)	The active group (n = 184) received a vitamin D and leucine-enriched whey protein nutritional supplement to consume twice daily for 13 weeks. The control group (n = 196) received an isocaloric control product to consume twice daily for 13 weeks.	The 13-week intervention of a vitamin D and leucine-enriched whey protein oral nutritional supplement resulted in improvements in muscle mass and lower-extremity function among sarcopenic elderly adults.
Verreijen et al [33]	Obese elderly adults (63 ± 5.6 years; body mass index, 33 ± 4.4 kg/m^2^, n = 80)	All subjects followed a hypocaloric diet (-600 kcal/day) and performed resistance training 3 times/week. A high whey protein-, leucine-, and vitamin D-enriched supplement including a mix of other macro- and micronutrients (150 kcal, 21 g protein; 10 times/week) or an isocaloric control.	A high whey protein-, leucine-, and vitamin D-enriched supplement compared with isocaloric control preserved muscle mass.
Hsieh et al [34]	Bed-ridden elderly nursing home residents receiving tube feeding (n = 79)	Subjects were randomly assigned to HMB (n = 39, 2 g/day) or control group (n = 40) for 4 weeks.	HMB supplementation for 2 - 4 weeks could reduce muscle breakdown in bed-ridden elderly nursing home residents receiving tube feeding.
Flakoll et al [35]	Women (mean 76.7 years, n = 50)	Subjects were randomized to a placebo group (n = 23) or an experimental treatment group (2 g HMB, 5 g arginine, and 1.5 g lysine daily; n = 27) for 12 weeks.	Daily supplementation of HMB, arginine, and lysine for 12 weeks positively altered measurements of muscle functionality, strength, fat-free mass, and protein synthesis.
Baier et al [36]	Elderly (76 ± 1.6 years) women (n = 39) and men (n = 38)	Participants were randomly assigned to either an isonitrogenous control-supplement (n = 37) or a treatment-supplement (HMB/arginine/lysine) (n = 40) for 1 year.	Consumption of a simple amino acid-related cocktail increased protein turnover and LM in elderly individuals in a year-long study.

AAS: amino acid supplementation; CMR: competitive meal replacement; EAA: essential amino acids; EAAMR: whey protein + essential amino acid meal replacement; HE: health education; HMB: beta-hydroxy-beta-methylbutyrate; LM: lean mass.

One hundred fifty-five sarcopenic women aged 75 and older were randomly assigned to EX and amino acid supplementation, EX, amino acid supplementation, or health education. EX and amino acid supplementation together was effective in enhancing not only muscle strength, but also combined variables of muscle mass and walking speed in sarcopenic women [22]. Daily ingestion of 15 g essential amino acids (EAA) improved LM and basal muscle protein synthesis in elderly individuals [23]. Ingestion of 8 g of EAA snacks twice a day, significantly increased LM after 6 months and more consistently after 18 months [24]. An oral amino acid supplement improved ambulatory capacity and maximal isometric muscle strength in elderly subjects [25]. Supplementation of the diet with EAA plus arginine also improved LM, strength and physical function compared to baseline values in glucose intolerant elderly individuals [26]. Coker et al recruited and randomized 12 elderly individuals to an 8-week, CR diet utilizing equivalent caloric meal replacements (800 kcal/day): 1) whey protein + EAA meal replacement (EAAMR) or 2) competitive meal replacement (CMR) in conjunction with 400 kcal of solid food that totaled 1,200 kcal/day designed to induce 7% weight loss [27]. Both groups lost about 7% of total body weight. While EAAMR did not promote a significant preservation of LM, the reduction in adipose tissue was greater in EAAMR compared to CMR. These results indicate that quality of protein and energy intake are associated with prevention of sarcopenia.

Aging is associated with changes in the muscle protein metabolism response to a meal, due to alterations in the response to endogenous hormones. The older muscle is still able to respond to amino acids such as leucine, leucine initiates mRNA translation, which is still present in elderly people [37]. Long-term EAA supplementation including excess leucine may be a useful tool for the prevention and treatment of sarcopenia [37]. Beta-hydroxy-beta-methylbutyrate (HMB), a metabolite of the branched-chain amino acid leucine, has been investigated as a potential supplement to improve muscle quality [38].

Bukhari et al performed a single-dose administration (whey protein or novel low-dose leucine-enriched EAA (3 g, 40% leucine)) [28]. However, they did not show the difference in muscle anabolism between whey protein and novel low-dose leucine-enriched EAA by a single-dose administration [28].

Vitamin D has been known to have a role in skeletal muscle health [39]. Further, regular EX is the strategy found to consistently prevent frailty and improve sarcopenia and physical function in elderly adults [40]. Resistance EX training is effective in increasing muscle mass and strength [40].

The supplementation of vitamin D alone provided significant protective effect against the occurrence of sarcopenia and significant increases in muscle strength in postmenopausal women [29]. In a 2 × 2 factorial-design trial, subjects were randomly assigned either to a whole-body vibration (WBV) or a no-training group, receiving either a conventional dose (880 IU/day) or a high dose (1,600 IU/day) of vitamin D [30]. A higher dose of vitamin D did not provide additional musculoskeletal benefit compared with conventional doses.

A protein-enriched diet equivalent to 1.3 g/kg/day achieved through lean red meat is safe and effective for enhancing the effects of progressive resistance training on LM and muscle strength [31]. The 13-week intervention of a vitamin D and leucine-enriched whey protein oral nutritional supplement resulted in improvements in muscle mass and lower-extremity function among sarcopenic elderly adults [32]. A high whey protein-, leucine-, and vitamin D-enriched supplement preserved muscle mass during intentional weight loss in obese elderly adults [33].

HMB supplementation for 2 - 4 weeks could reduce muscle breakdown in bed-ridden elderly nursing home residents receiving tube feeding [34]. Daily supplementation of HMB, arginine, and lysine for 12 weeks beneficially altered measurements of muscle functionality, strength, FFM, and protein synthesis [35]. Consumption of a simple amino acid-related cocktail (HMB, arginine and lysine) increased protein turnover and LM in elderly individuals in a year-long study [36].

## Conclusion

I reviewed published articles about the effects of nutritional factors on sarcopenia. Epidemiological studies suggested that a low protein intake is associated with sarcopenia. The effectiveness and safety of ER for sarcopenic obesity remains unclear. As optimal dietary protein intake, daily 1.0 - 1.2 g/kg with an optimal repartition over each daily meal or 25 - 30 g of high quality protein per meal were recommended to prevent sarcopenia, which was supported by some observational studies. Cheese and milk protein, EAA, leucine, HMB and vitamin D have been investigated as a potential supplement to improve muscle quality in sarcopenic elderly people. Some studies showed the effectiveness of these nutritional factors for sarcopenia. However, we should consider the appropriate concomitant energy intake, the combination of these factors and the combination with physical activities, to develop a better management for sarcopenia. Further studies, preferably with larger numbers of elderly subjects, will be needed.
